# High Luminous Efficacy Phosphor-Converted Mass-Produced White LEDs Achieved by AlN Prebuffer and Transitional-Refraction-Index Patterned Sapphire Substrate

**DOI:** 10.3390/nano12101638

**Published:** 2022-05-11

**Authors:** Shuo Zhang, Meng Liang, Yan Yan, Jinpeng Huang, Yan Li, Tao Feng, Xueliang Zhu, Zhicong Li, Chenke Xu, Junxi Wang, Jinmin Li, Zhiqiang Liu, Xiaoyan Yi

**Affiliations:** 1 Research and Development Center for Solid State Lighting, Institute of Semiconductors, Chinese Academy of Sciences, Beijing 100083, China; zshuo@semi.ac.cn (S.Z.); liangmeng@semi.ac.cn (M.L.); yanyan19@semi.ac.cn (Y.Y.); jinpenghuang@csu.edu.cn (J.H.); yanli7@semi.ac.cn (Y.L.); ftao18@semi.ac.cn (T.F.); lizc@semi.ac.cn (Z.L.); jxwang@red.semi.ac.cn (J.W.); jmli@red.semi.ac.cn (J.L.); 2 Center of Materials Science and Optoelectronics Engineering, University of Chinese Academy of Sciences, Beijing 100049, China; 3 Xiamen San’an Optoelectronic Technology Co., Ltd., Xiamen 361009, China; zhuxueliang@sanan-e.com (X.Z.); troy@sanan-e.com (C.X.); 4 Yangzhou Zhongke Semiconductor Lighting Company, Yangzhou 225101, China; 5 Beijing Engineering Research Center for the 3rd Generation Semiconductor Materials and Application, Beijing 100083, China

**Keywords:** light-emitting diodes, AlN prebuffer, transitional-refraction-index patterned sapphire substrate, light output power, luminous efficacy

## Abstract

Constant advance in improving the luminous efficacy (*η*_L_) of nitride-based light-emitting diodes (LEDs) plays a critical role for saving measurable amounts of energy. Further development is motivated to approach the efficiency limit for this material system while reducing the costs. In this work, strategies of using thin AlN prebuffer and transitional-refraction-index patterned sapphire substrate (TPSS) were proposed, which pushed up the efficiency of white LEDs (WLEDs). The AlN prebuffer was obtained through physical vapor deposition (PVD) method and TPSS was fabricated by dry-etched periodic silica arrays covered on sapphire. Devices in mass production confirmed that PVD AlN prebuffer was able to improve the light output power (*φ*_e_) of blue LEDs (BLEDs) by 2.53% while increasing the productivity by ~8% through shortening the growth time. Additionally, BLEDs on TPSS exhibited an enhanced top *η*_ext_ of 5.65% in contrast to BLEDs on the conventional PSS through Monte Carlo ray-tracing simulation. Consequently, *φ*_e_ of BLEDs was experimentally enhanced by 10% at an injected current density (*J*_in_) of 40 A/cm^2^. A peak *η*_L_ of 295.2 lm/W at a *J*_in_ of 0.9 A/cm^2^ and the representative *η*_L_ of 282.4 lm/W at a *J*_in_ of 5.6 A/cm^2^ for phosphor-converted WLEDs were achieved at a correlated color temperature of 4592 K.

## 1. Introduction

A fundamental transformation of lighting technology was triggered in the early 1990s with the emerging InGaN-based light-emitting diodes (LEDs). Nowadays LEDs are widely used in the fields of indoor and outdoor lighting [1,2,3], full-color displays [4,5,6], and applications beyond lighting [7,8,9,10] because of the inherent advantages of nitride LEDs, including high efficiency, robustness, low heat output, nontoxicity, and long life span [11,12,13]. Assuming that solid-state lighting sources could replace conventional light sources worldwide, energy consumption would be remarkably reduced. This is equivalent to approximately 230 typical 500-MW coal plants or 200 million tons of greenhouse gas emissions [12]. White LEDs (WLEDs) are usually realized by two strategies: one is phosphor-converted LEDs (PC-LEDs), which are achieved by mixing emission from blue LEDs (BLEDs) with the excited light from yellow phosphor [14,15]. Another one is multicolor LEDs, where several monochromatic LEDs are mixed to produce white light [16]. However, due to the lack of high-efficiency InGaN-based long-wavelength LEDs, this strategy remains difficult to implement. At present, PC-LEDs are the most mainstream technology for various applications.

As predicted, the theoretical luminous efficacy (*η*_L_) limit of PC-WLEDs is ~350 lm/W [17]. Additionally, its practical limit is forecasted to be 255 lm/W [18]. In this regard, Cree Inc. first reported that the *η*_L_ of WLEDs on SiC (probably) exceeded 300 lm/W, details of which were not revealed [19]. For many decades, many efforts have been taken to improve the internal quantum efficiency (*η*_int_), light extraction efficiency (*η*_ext_), and conversion efficiency of phosphor (*η*_phos_), which improves the overall *η*_L_ of PC-LEDs. For *η*_int_ and *η*_ext_, the typical techniques focus on (a) improving the crystalline quality of epilayers by using low-temperature (LT) buffer layers [20], epitaxial lateral overgrowth [21,22], and patterned sapphire substrate (PSS) [23,24]; (b) optimization of the device structures including multiple quantum wells (MQWs) with stronger radiative recombination [25,26], electron blocking layer (EBL) suppressing current leakage [27,28], and *p*-type layer with high hole concentration [29]; and (c) high *η*_ext_ design, involving PSS [23,24], microstructured air cavities [30], and mirror reflectors [31,32]. In terms of *η*_phos_, multiple types of phosphors have been introduced to improve color rendering index while manifesting high efficiency [33,34]. Nevertheless, there is still great room for efficiency improvement that can yield additional substantial energy savings.

In this work, we fabricated high-efficiency WLEDs by employing physical vapor deposition (PVD) AlN prebuffer and transitional-refraction-index patterned sapphire substrate (TPSS) technologies. Mass production device results demonstrated an increase of productivity by ~8%, with an enhancement in the light output power (*φ*_e_) of BLEDs from 158 to 162 mW at an injected current of 120 mA. Simulation results showed that the BLEDs grown on TPSS enabled a top *η*_ext_ improvement of 5.65%. Simultaneously, *φ*_e_ is experimentally enhanced by 10% at an injected current density (*J*_in_) of 40 A/cm^2^. Consequently, the PC-WLEDs exhibited a peak *η*_L_ of 295.2 lm/W at a *J*_in_ of 0.9 A/cm^2^, which provided effective strategies for achieving super high-efficiency solid-state lighting.

## 2. Materials and Methods

**Figure 1:**
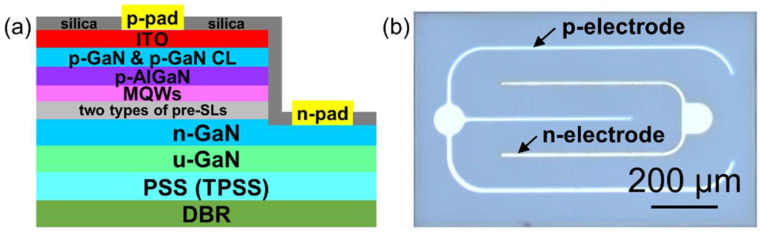
(**a**) Schematic diagram of the LED structure from a cross-sectional view including epilayers and technical processes; (**b**) Optical photo of the as-fabricated lateral LED chip.

The GaN-based epitaxial layers were grown on two types of substrates: (a) 4-inch PSS and (b) 4-inch TPSS. Both pattern arrays were achieved by standard photo-lithography and dry etching. All epilayers of the BLED structure were synthesized by metal-organic chemical vapor deposition (MOCVD, Veeco, k700). During the MOCVD process, Trimethylgallium (TMGa) or triethygallium (TEGa), trimethylaluminum (TMAl), trimethylindium (TMIn), NH_3_, silane (SiH_4_), and magnesocene (Cp_2_Mg) were adopted as Ga, Al, In, N precursors, *n*-doped and *p*-doped sources, respectively. The buffer layer growth process will be discussed in detail in subsequent sections. As shown in Figure 1a, the MOCVD process consists of a 3.5 μm undoped GaN (*u*-GaN) layer, a 1.5 μm n-doped GaN (*n*-GaN) layer with Si-doped concentration of ~2.5 × 10^19^ cm^−3^, two types of InGaN/GaN pre-superlattices (pre-SLs) defined as pre-SLs-I and pre-SLs-II, MQWs, a *p*-doped AlGaN electronic barrier layer (EBL), a *p*-doped GaN (*p*-GaN) layer with Mg-doped concentration of ~3.6 × 10^19^ cm^−3^, and a *p*-GaN contact layer (CL). The detailed growth conditions of the above processes are summarized in Table 1. Finally, the *p*-GaN layer and *p*-GaN CL were annealed in-situ at 720 °C to form good ohmic contacts. The design of pre-SL layers aims to both relax the stress in MQWs and improve the horizontal distribution of injected electrons, and then enhances the *η*_L_ [35]. Al-composition-graded *p*-AlGaN EBL contributes to blocking electrons escaping from the active region [36].

A layer of indium tin oxide film was deposited on *p*-GaN CL with a thickness of 50 nm as the current spreading layer, and then annealed at 550 °C for 30 min in N_2_ atmosphere. The mesa was achieved by standard photolithography and the inductively coupled plasma etching process. Cr/Al/Ti/Au (50/1700/40/200 nm) layers were deposited through electron beam evaporation to act as *p*- and *n*- contact electrodes, and then annealed at 250 °C for 15 min. The devices were passivated by a layer of SiO_2_ (225 nm) through plasma-enhanced chemical vapor deposition. Afterwards, 40 pairs of Ti_2_O_5_/SiO_2_ (46.4/76.8 nm) distributed Bragg reflectors (DBRs) were coated on the backside of the wafer after grinding and polishing processes. The BLED chips were separated by wafer laser dicing. The devices were mounted on the alumina substrates and wire-bonded followed by covering yellow phosphors. Figure 1a illustrates a cross-sectional schematic diagram of an LED chip. An optical photo of a chip (22 × 40 mil^2^) is presented in Figure 1b. The optical and electronic performance of these two types of BLEDs were tested by the integrating sphere (Everfine).

To precisely evaluate the influence of PVD AlN prebuffer on the device performance, a mass production test was run over twenty different MOCVD chambers for half a month. Ten wafers were grown in each MOCVD on a daily basis, using conventional LT GaN buffer layer (defined as W/O AlN, 540 °C LT buffer layer for five wafers) and PVD AlN prebuffer (800 °C HT buffer layer for five wafers). The lateral LEDs were processed for all the wafers to fabricate BLED chips with dimensions of 9 × 28 mil^2^. The *φ*_e_ at a current of 120 mA was obtained through chip on tape (COT) measurement.

A simulation using the Monte Carlo ray-tracing method was performed to evaluate the optical characteristics of BLEDs on flat sapphire substrate technology (BLED-FSS), PSS (BLED-PSS), and TPSS (BLED-TPSS) [37]. The size of the chip was set to 60 µm^2^. A Lambertian light emission from the active area with a wavelength of 450 nm and *φ*_e_ of 1 W (P_total_ = 1 W) was adopted. The ray incidence on the upper hemisphere surface is collected. A perfect absorber with a radius of 1 mm was designed to count the output *φ*_e_ for the three models (see Figure S1a–c). The top *η*_ext_ is defined as the ratio of *φ*_e_ that escapes into the hemisphere surface (P_out_) to the source power (P_total_).

The morphology of the substrate and epilayers was characterized by scanning electron microscopy (SEM, Hitachi, Tokyo, Japan; operated at 4.4 kV). Dislocations were analyzed through high-resolution transmission electron microscopy (HRTEM, JEM-F200), and X-ray diffraction spectroscopy (XRD, Bede D1, Durham, UK; operated at 40 kV, 35 mA). The reflectance of the epilayers was measured through a spectrophotometer (Hitachi, UH4150, Tokyo, Japan), with the incident light wavelengths ranging from ultraviolet to near-infrared.

## 3. Results and Discussion

### 3.1. PVD AlN Prebuffer Layer

**Figure 2:**
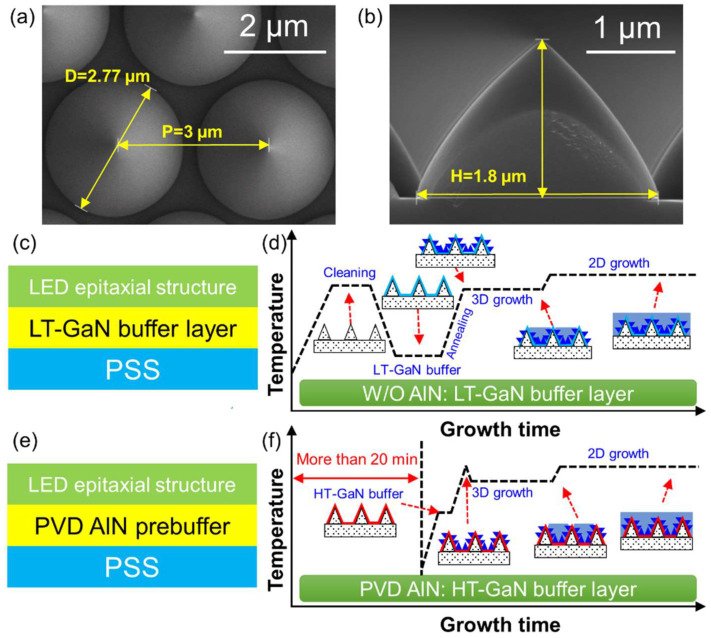
(**a**) Plane-view and (**b**) cross-sectional SEM images of PSS; (**c**) schematic diagram and (**d**) schematic curve of temperature transients of LED epitaxial structure with LT-GaN buffer layer; (**e**) schematic diagram and (**f**) schematic curve of temperature transients of LED epitaxial structure with PVD AlN prebuffer layer.

In this part, PVD AlN prebuffer layer, in contrast to W/O AlN for BLED epitaxial structure, was demonstrated in terms of the growth time, material quality, and *φ*_e_ in the mass production. Because of the large lattice mismatch (~16%) and thermal mismatch between GaN film and (0001) sapphire substrate [38], improving the material quality is crucial for fabrication of high efficiency nitride-based devices. Disruptive breakthroughs of high-quality GaN films originated from the development of LT GaN or AlN buffer layers [20,39]. The LED epitaxial structure was grown on 4-inch PSS, yielding periodically arrayed cone-shaped sapphire substrates. The height and base diameter of the cone were 1.8 µm and 2.77 µm (H = 1.8 µm, D = 2.77 µm), respectively, and the period was 3 µm (*p* = 3 µm) (see Figure 2a,b). The cone-shaped array exhibited a smooth sidewall and good periodicity. This is beneficial to the uniform gas flow field distribution of the reaction chamber, and then ensures the uniformity of electrical and luminescence properties of LED devices. Epitaxy processes of LED samples using W/O AlN and PVD AlN prebuffer during the MOCVD process were compared. For W/O AlN, Figure 2c,d show schematic diagrams of the epitaxial structure and curves of temperature transients during the MOCVD process. In detail, PSS first underwent a heating and high-temperature (HT) cleaning process at 1050 °C. Then, the LT-GaN layer was grown at 540 °C for 4 min through a cooling procedure maintained for approximately 13 min. Subsequently, after a 9 min heating ramp, three-dimensional (3D) island growth and two-dimensional (2D) lateral growth were implemented in order.

The AlN prebuffer layer with a thickness of ~20 nm was deposited on PSS by PVD (Endura 300, Applied Materials, Inc., Santa Clara, CA, USA). Afterward, the AlN/PSS templates were loaded into the MOCVD reactor for the growth of LED epilayers. The corresponding schematic diagram of the epitaxial structure and trace-record curve of temperature transients were depicted in Figure 2e,f. An HT buffer layer was directly deposited on the template followed by the 3D island and 2D lateral deposition process after a heating ramp. In this case, the complicated heating and cooling ramp lasting of the LT buffer were simplified. This shortened the growth time by more than 20 min and increased the productivity by ~8%. Considering the high operating cost of MOCVD, the introduction of PVD AlN prebuffer layer in nitride epitaxy provides a new thought for the high-efficiency and low-cost growth of nitrides, which is meaningful in industrialized mass production.

**Figure 3:**
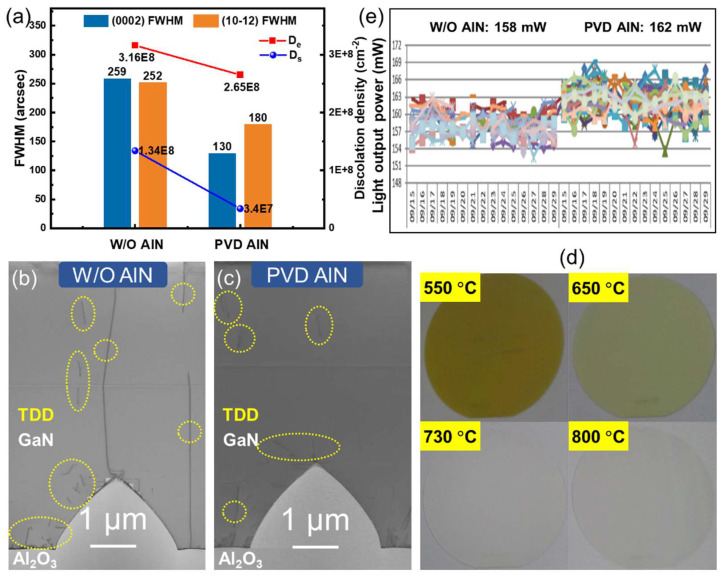
(**a**) The mean FWHM values of (0002) and (10–12) rocking curves of epitaxial wafers utilizing W/O AlN and PVD AlN prebuffer, D_s_: screw TDD, D_e_: edge TDD; TEM images of BLED epilayers using (**b**) W/O AlN, (**c**) PVD AlN prebuffer; (**d**) The optical photo of the wafers, in which the GaN buffer layer was grown at 550, 650, 730, and 800 °C using PVD AlN prebuffer. (**e**) *φ*_e_ data logging results of mass production wafers using W/O AlN and PVD AlN prebuffer for half a month.

Consequently, a trial-produced BLED film on a 4-inch PSS comparing the W/O AlN and PVD AlN prebuffer was executed to investigate the material quality and LED characteristics. The mean full width at half maximum (FWHM) values of the (0002) and (10–12) rocking curves were measured as 259, 252 arcsec for W/O AlN, and 130, 180 arcsec for PVD AlN prebuffer, respectively (see Figure 3a). Therefore, the screw threading dislocation density (TDD) and edge TDD are derived as: (a) 1.34 × 10^8^ and 3.16 × 10^8^ cm^−3^ for W/O AlN, and (b) 3.4 × 10^7^ and 2.65 × 10^8^ cm^−3^ for PVD AlN prebuffer [40], which indicates that the screw TDD drops by approximately an order of magnitude and the edge TDD decreases slightly. Cross-sectional TEM images of LED epilayers are shown in Figure 3b,c, which verifies that the TDD is effectively reduced for BLEDs with the PVD AlN prebuffer. Improvement of crystalline quality could benefit the *η*_int_ of the LED structure via suppressing the Shockley–Read–Hall recombination process. [41]. As discussed, an HT buffer layer was implemented on PVD AlN prebuffer layer. Therefore, different growth temperatures for HT buffer layer were executed in MOCVD. From Figure 3d, it can be observed that as the temperature increases, the color of the wafer gradually fades from dark yellow to nearly transparent, suggesting the reduction of defect densities under HT conditions. Therefore, the LT GaN buffer layer absorbs more blue light emitted from MQWs because of the defect-related photoluminescence when the LED device is operated. This is bad for enhancing *η*_ext_ [42].

The average *φ*_e_ data of each batch of wafers in different reactors were recorded daily (see Figure 3e). The average *φ*_e_ of all wafers over half a month duration test for W/O AlN and PVD AlN prebuffer are around 158 and 162 mW, respectively. This suggests that the *φ*_e_ of BLED has been increased by 2.53%, in which accidental factors are excluded based on the statistics of the quantity. In summary, the development of PVD AlN prebuffer not only improves the production efficiency, but also optimizes the working performance of LED devices.

### 3.2. TPSS Technology

**Figure 4:**
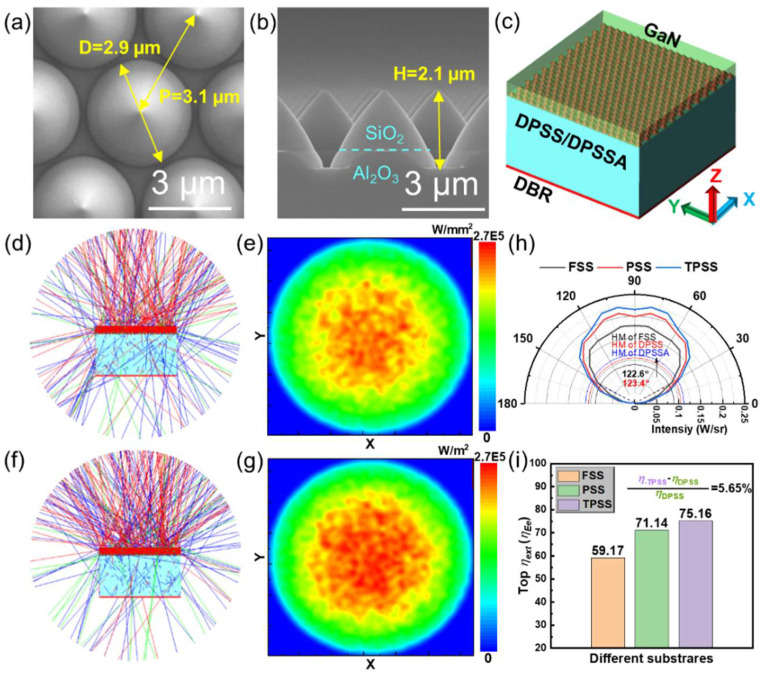
(**a**) Plane-view and (**b**) cross-sectional SEM images of TPSS; (**c**) The simulated modeling of BLED-PSS or BLED-TPSS; (**d**) Cross-sectional ray-tracing and (**e**) radiation patterns on hemisphere surface of BLED-PSS; (**f**) Cross-sectional ray-tracing and (**g**) radiation patterns on hemisphere surface of BLED-TPSS; (**h**) Simulated far-field radiation patterns and (**i**) calculated top *η*_ext_ of BLED-FSS, BLED-PSS, and BLED-TPSS.

In this section, a novel TPSS technology was introduced in the BLED structure compared with conventional PSS technology mainly for the promotion of *η*_ext_ through pilot study. For TPSS, the cone material is replaced from sapphire to silica, with a size of H = 2.1 µm, D = 2.9 µm, and periodicity of *p* = 3.1 µm, which is the same as the features of PSS (see Figure 4a,b). These two technologies combined with PVD AlN prebuffer were adopted to grow LED epitaxial structures, and the MQW layer was located approximately 100 nm from the top.

**Figure 5:**
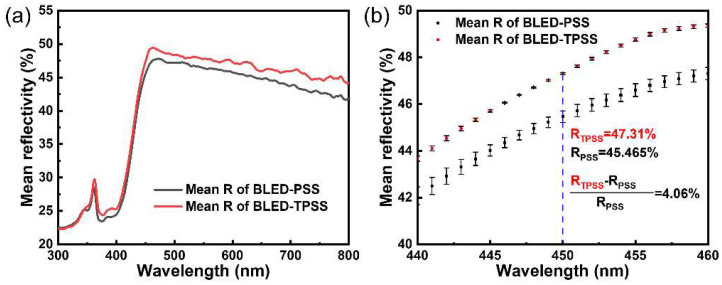
(**a**) Reflection spectra of BLED-PSS and BLED-TPSS wafers with incident wavelengths from 300 nm to 800 nm; (**b**) Zoomed reflection spectra at incident wavelengths around 450 nm.

To save computational memory, the BLED modeling was simplified as four layers, including an active region, GaN epilayer, substrates layer, and DBR layer with a reflectivity of 99% from top to bottom (see Figure 4c). The cross-sectional ray-tracing of BLED-FSS, BLED-PSS, BLED-TPSS, and corresponding radiation patterns on the hemisphere surface are sketched in Figure S1d–e and Figure 4d–g. It is observed that the BLED-PSS and BLED-TPSS exhibit significantly enhanced light emissions in all directions compared with the BLED-FSS. It is worth mentioning that BLED-TPSS demonstrates a stronger upward and weaker downward light distribution than BLED-PSS. The intensity mapping of the *φ*_e_ density in Figure 4g for BLED-TPSS is higher than that in Figure 4e for BLED-PSS. This is consistent with the results of ray-tracing. Figure 4h presents the simulated far-field radiation patterns of BLEDs on different substrates. We assume that the pattern is an ideal symmetrical distribution and the result in the range of 0–90° is simulated. For the emission intensity, that of BLED-TPSS is the highest at almost all launch angles. In terms of the half-value (HV) angle, BLED-FSS, BLED-PSS, and BLED-TPSS are 122.6°, 123.4°, and 123.4°, respectively. This means that BLED-PSS and BLED-TPSS have almost the same emission HV angle, which is slightly wider than BLED-FSS. The top *η*_ext_ values of the three-type LEDs are 59.17%, 71.14%, and 75.16%, as shown in Figure 4i. Compared with BLED-PSS, the top *η*_ext_ of BLED-TPSS is increased by 5.65%. Although a small value appears, it is a large breakthrough for industrial production. As the reflectivity of the epitaxial structure is an important factor for LED light extraction, the reflectivity of BLED-PSS and BLED-TPSS wafers was measured to investigate the collimated *η*_ext_ effect (see Figure 5a,b). The reflectivity of BLED-TPSS is slightly higher than that of BLED-PSS in the range of incident light wavelengths from ultraviolet to near-infrared. In particular, a significant improvement of 4.06% at 450 nm is observed, explaining the stronger *η*_ext_ intensity along the axial direction, which coincides with the simulated results. In addition, during the epitaxial process, AlN/TPSS could improve the interfacial growth front compared to AlN/PSS (see Figure S2), with detailed discussion in Supplementary Materials. The thickness of epitaxial LED structure on both AlN/PSS and AlN/TPSS is around 5.75 µm (see Figure S3).

### 3.3. High Light Output Power BLED

**Figure 6:**
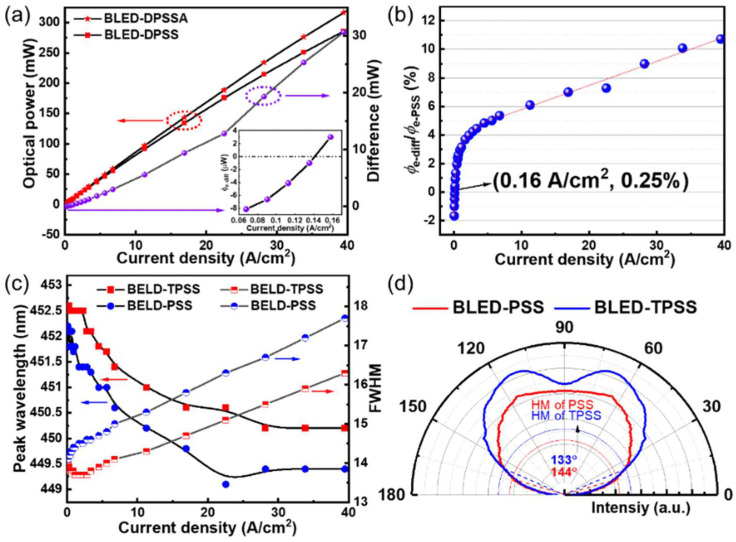
(**a**) *φ*_e_ and its difference, (**b**) the *φ*_e_ enhancement, (**c**) λ_p_ and FWHM of BLED-PSS and BLED-TPSS chips as functions of *J*_in_, the inset of (**a**) is an expansion of *φ*_e-diff_ in the range of *J*_in_ less than 0.16 A/cm^2^; (**d**) Experimentally measured far-field radiation patterns of BLED-PSS and BLED-TPSS.

The BLED devices were fabricated with dimensions of 22 × 40 mil^2^, using PSS and TPSS combined with PVD-AlN prebuffer. *φ*_e_ of BLED-TPSS (*φ*_e-TPSS_) and *φ*_e_ of BLED-PSS (*φ*_e-PSS_) versus *J*_in_ are sketched in Figure 6a. The relative enhancement of *φ*_e_ (*φ*_e-relative_) is described as the ratio of difference *φ*_e_ (*φ*_e-diff_) to *φ*_e-DPSS_, expressed as *φ*_e-diff_/*φ*_e-PSS_ (see Figure 6b). It can be seen that *φ*_e-TPSS_ is slightly lower than *φ*_e-PSS_ when *J*_in_ is less than 0.16 A/cm^2^. However, *φ*_e-TPSS_ is higher in a large current density range greater than 0.16 A/cm^2^. As *J*_in_ increases, *φ*_e-relative_ shows logarithmic growth first when *J*_in_ is less than 6.8 A/cm^2^, and then exhibits a linear increase when *J*_in_ is greater than 6.8 A/cm^2^. The *φ*_e-relative_ has exceeded 10% at a *J*_in_ of 40 A/cm^2^.

The peak wavelength (λ_p_) and FWHM of the electroluminescence (EL) spectra are presented in Figure 6c. The λ_p_ of both BLED-PSS and BLED-TPSS is located at approximately 450 nm, corresponding to the radiation recombination of electron–hole pairs of blue emission. λ_p_ blueshifts from 452.2 to 449.4 nm for BLED-PSS, and 452.6 to 450.2 nm for BLED-TPSS as *J*_in_ increases from 0.07 to 39.5 A/cm^2^. In this case, a quantum-confined Stark effect (QCSE) is generated within the MQW region at no *J*_in_ caused by spontaneous and built-in piezoelectric fields, especially for the LED structure [43], which will reduce the overlap integral of electron/hole wave-functions. As a result, the charge screening and band filling effects would gradually weaken the QCSE as *J*_in_ increases, subsequently leading to a blueshift in the λ_p_. The FWHM of both LEDs behaves faintly larger when *J*_in_ changes from 0.07 to 40 A/cm^2^. This is ascribed to the native fluctuation of In composition in MQWs, and more electron–hole pairs would be excited as *J*_in_ increases [44]. Far-field radiation patterns of BLED-PSS and BLED-TPSS in the range of 0–90° are experimentally measured (see Figure 6d). Compared to BLED-PSS, BLED-TPSS exhibits a higher relative intensity, which is consistent with the simulation results. However, the deviation is that the HVs of BLED-PSS and BLED-TPSS are larger than the simulated results, in which the experimental results are affected by many processes, such as dicing, gold wires, and package substrate. Additionally, BLED-TPSS has a more convergent emission pattern with an HV 11° lower than that of BLED-PSS.

### 3.4. High Luminous Efficacy WLED

**Figure 7:**
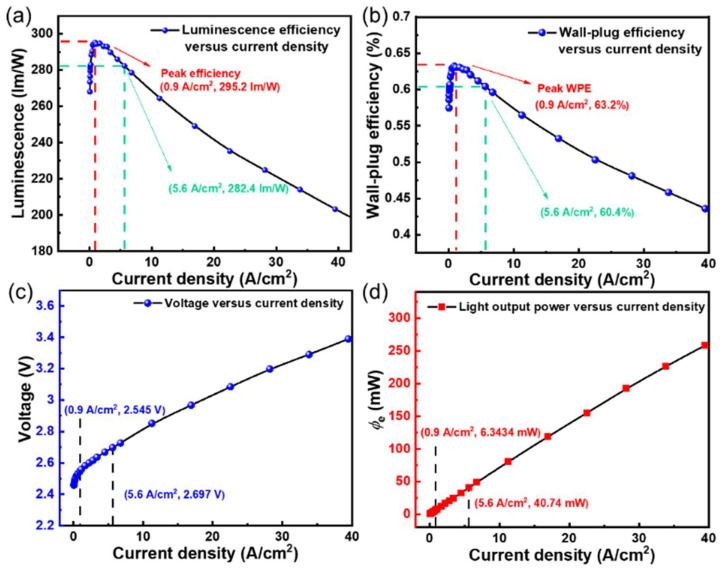
(**a**) *η*_L_ as a function of *J*_in_ for WLEDs; (**b**) WPE as functions of Jin for white LEDs; (**c**) *J*_in_ and (**d**) *φ*_e_ dependence of operating voltage for WLEDs.

We fabricated WBEDs by incorporating BLEDs and yellow phosphor through a PC-LED package strategy. The *J*_in_ dependence of *η*_L_, wall-plug efficiency (WPE), operated voltage, and *φ*_e_ are shown in Figure 7. The peak *η*_L_ and peak WPE reach maxima of 295.2 lm/W and 63.2%, respectively, at a *J*_in_ of 0.9 A/cm^2^. It is worth noting that the *η*_L_ and WPE retained 282.4 lm/W and 60.4% at a *J*_in_ of 5.6 A/cm^2^, respectively. The *η*_L_ and WPE decrease as *J*_in_ (greater than 0.9 A/cm^2^) increases, which is the common droop effect for LED devices. It may originate from Auger recombination, carrier leakage, carrier localization, and other factors [45,46]. 

The *J*-V curve of the WLED demonstrates good rectification characteristics with two representative points of (0.9 A/cm^2^, 2.55 V), (5.6 A/cm^2^, 2.7 V). The *φ*_e_ shows a linear increase with increasing *J*_in_ and reaches 40.74 mW at a *J*_in_ of 5.6 A/cm^2^. The correlated color temperature (CCT) was measured as 4592 K, where neutral white light was obtained. This work has significantly enhanced the brightness of WLEDs, which is expected to promote the applications of superior solid-state lighting in general lighting, high-power lighting, and display fields in the future.

## 4. Conclusions

In summary, high-efficiency WLEDs have been demonstrated by adopting PVD AlN prebuffer and TPSS technology. The *η*_L_ reaches 295.2 lm/W and 282.4 lm/W at *J*_in_ of 0.9 A/cm^2^ and 5.6 A/cm^2^, respectively. The PVD AlN prebuffer enables the screw TDD to drop by approximately an order of magnitude from 1.34 × 10^8^ to 3.4 × 10^7^ cm^−3^. It then enhances the *φ*_e_ of BLEDs by 2.53% via batch data statistics, while increasing the productivity of ~8%. Monte Carlo ray-tracing simulations show that the top *η*_ext_ has been increased from 71.14% for BLED-PSS to 75.16% for BLED-TPSS. The reflectivity of light peaking at 450 nm for BLED-TPSS is enhanced by 4.06%, and the *φ*_e_ is promoted by 10% at a *J*_in_ of 40 A/cm^2^. The goal of this work is to reduce energy waste with technological innovations in solid-state lighting to achieve higher quantum efficiency. Future work will focus on the mature mass production and commercialization of the strategies proposed in this work.

## Figures and Tables

**Table 1:** The growth conditions of the epitaxial structure in the MOCVD process.

Step	Temperature (°C)	Time (min)	NH_3_ (slm)	TMGa (sccm)	TEGa (sccm)	TMAl (sccm)	TMIn (sccm)	SiH_4_ (sccm)	Cp_2_Mg (sccm)
*u*-GaN	1040	53	146	1490	/	/	/	/	/
*n*-GaN	1070	23	112	740	/	/	/	50.6	/
InGaN/GaN pre-SLs-I (LOOP 3)	860	2	90	/	490	/	1280	/	/
	860	4	90	/	390	/	/	/	/
InGaN/GaN pre-SLs-II (LOOP 4)	800	2	90		315	/	1125	/	/
	885	4	90		1010	/	/	/	/
InGaN/GaN MQWs (LOOP 10)	760	4	90	/	315	/	1125	/	/
	885	8	90	/	1010	/	/	/	/
*p*-AlGaN EBL	950	5	22	150	/	0–140	/	/	360
*p*-GaN	950	10	112	180	/	/	/	/	765
*p*-GaN CL	680	1.5	90	/	315	/	790	/	394

## Data Availability

The data presented in this study are available on request from the corresponding author.

## Supplementary Materials

The following supporting information can be downloaded at: https://www.mdpi.com/article/10.3390/nano12101638/s1, Figure S1. Ray-tracing results of BLED on (a) FSS, (b) PSS, (c) TPSS using hemispherical perfect absorber model; (d) Cross-sectional ray-tracing and, (e) radiation patterns on hemisphere surface of BLED on FSS. Figure S2. (a) Cross-section and (b) plane-view SEM images of GaN 3D growth on AlN/PSS template; (c) Cross-section and (d) plane-view SEM images of GaN 3D growth on AlN/TPSS template. Figure S3. Cross-section SEM images of LED epitaxial structure on (a) AlN/PSS template and (b) AlN/TPSS template.
