# Supplementary material for: High Luminous Efficacy Phosphor-Converted Mass-Produced White LEDs Achieved by AlN Prebuffer and Transitional-Refraction-Index Patterned Sapphire Substrate
Source: Nanomaterials (Basel). 2022 May 11;12(10):1638. doi: 10.3390/nano12101638 (PMC9147444; doi:10.3390/nano12101638)
Supplement: Supplementary file 1 [file nanomaterials-12-01638-s001.zip › nanomaterials-1700080-supplementary.pdf]

nanomaterials

Supplemental document

High Luminous Efficacy Phosphor-Converted Mass-Produced White LEDs Achieved by AlN Prebuffer and Transitional-Refraction-Index Patterned Sapphire Substrate

Shuo Zhang [1,2,5], Meng Liang [1,2,5], Yan Yan [1,2,5], Jinpeng Huang [1], Yan Li [1,2,5], Tao Feng [1,2,5], Xueliang Zhu [3], Zhicong Li [1,2,4], Chenke Xu [3], Junxi Wang [1,2,5], Jinmin Li [1,2,5], Zhiqiang Liu [1,2,5,]* and Xiaoyan Yi [1,2,5,]*

1 Research and Development Center for Solid State Lighting, Institute of Semiconductors, Chinese Academy of Sciences, Beijing 100083, China; zshuo@semi.ac.cn (S.Z.); liangmeng@semi.ac.cn (M.L.); yanyan19@semi.ac.cn (Y.Y.); jinpenghuang@csu.edu.cn (J.H.); yanli7@semi.ac.cn (Y.L.); ftao18@semi.ac.cn (T.F.); lizc@semi.ac.cn (Z.L.); jxwang@red.semi.ac.cn (J.W.); jmli@red.semi.ac.cn (J.L.)
2 Center of Materials Science and Optoelectronics Engineering, University of Chinese Academy of Sciences, Beijing 100049, China
3 Xiamen San'an Optoelectronic Technology Co., Ltd., Xiamen 361009, China; zhuxueliang@sanan-e.com (X.Z.); troy@sanan-e.com (C.X.)
4 Yangzhou Zhongke Semiconductor Lighting Company, Yangzhou 225101, China
5 Beijing Engineering Research Center for the 3rd Generation Semiconductor Materials and Application, Beijing 100083, China
* Correspondence: lzq@semi.ac.cn (Z.L.); spring@semi.ac.cn (X.Y.)

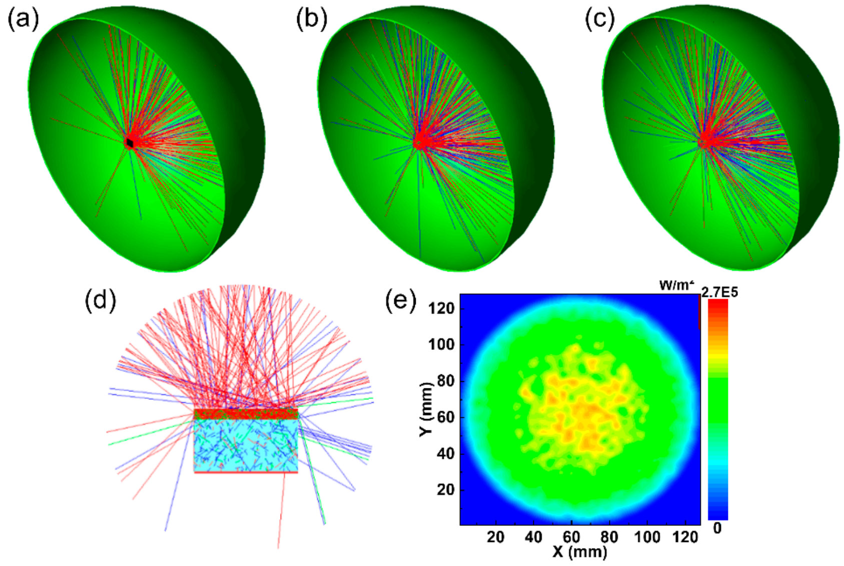

Figure S1. Ray-tracing results of BLED on (**a**) FSS, (**b**) PSS, (**c**) TPSS using hemispherical perfect absorber model; (**d**) Cross-sectional ray-tracing and, (**e**) radiation patterns on hemisphere surface of BLED on FSS.

Comparison for SEM images of 3D growth on AlN/PSS and AlN/TPSS is shown in Figure S2. It could be seen that irregular amorphous particles were deposited on sidewalls of PSS but not on sidewalls of TPSS. Uniform 3D growth of nitrides were observed in the middle of pyramid patterns for both AlN/PSS and AlN/TPSS. This indicates that AlN/TPSS could improve the interfacial growth front compared to AlN/PSS.

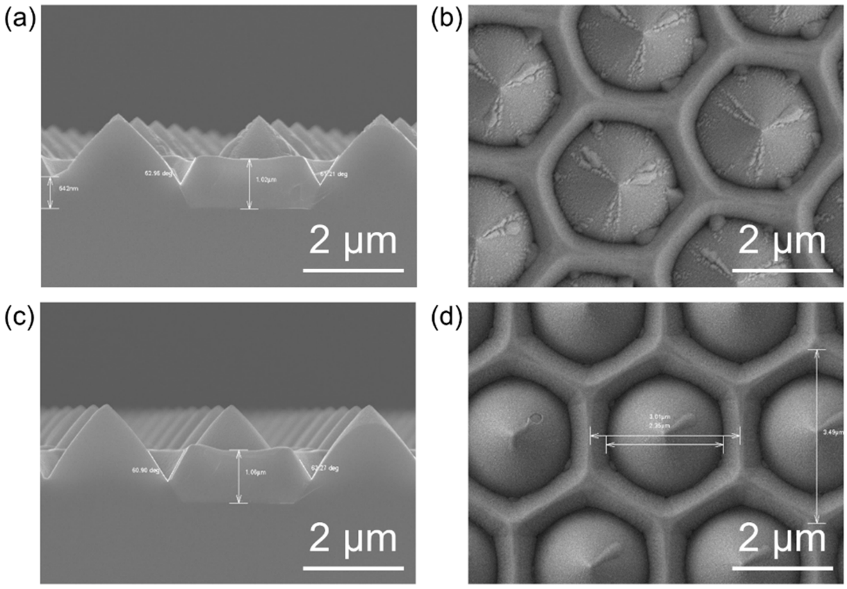

Figure S2. (**a**) Cross-section and (**b**) plane-view SEM images of GaN 3D growth on AlN/PSS template; (**c**) Cross-section and (**d**) plane-view SEM images of GaN 3D growth on AlN/TPSS template.

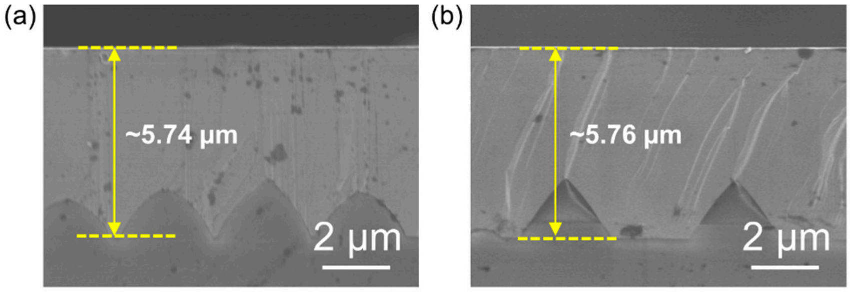

Figure S3. Cross-section SEM images of LED epitaxial structure on (**a**) AlN/PSS template and (**b**) AlN/TPSS template.
